# Markedly Elevated Aspartate Aminotransferase from Non-Hepatic Causes

**DOI:** 10.3390/jcm12010310

**Published:** 2022-12-30

**Authors:** Ji-Hee Han, Ji-Yoon Kwak, Sang-Soo Lee, Hyun-Gyu Kim, Hankyu Jeon, Ra-Ri Cha

**Affiliations:** 1 Department of Internal Medicine, Gyeongsang National University School of Medicine, Jinju 52727, Republic of Korea; 2 Department of Internal Medicine, Gyeongsang National University Changwon Hospital, Changwon 51472, Republic of Korea; 3 Institute of Health Sciences, Gyeongsang National University, Jinju 52727, Republic of Korea; 4 Department of Internal Medicine, Gyeongsang National University Hospital, Jinju 52727, Republic of Korea

**Keywords:** elevated aspartate aminotransferase, etiology, acute myocardial infarction, rhabdomyolysis, hemolysis

## Abstract

There have been no reports on mortality in patients with markedly elevated aspartate aminotransferase (AST) levels from non-hepatic causes to date. This study aimed to determine the etiologies of markedly elevated AST levels > 400 U/L due to non-hepatic causes and to investigate the factors associated with mortality in these cases. This retrospective study included 430 patients with AST levels > 400 U/L unrelated to liver disease at two centers between January 2010 and December 2021. Patients were classified into three groups according to etiology: skeletal muscle damage, cardiac muscle damage, and hematologic disorder. Binary logistic regression analysis was performed to evaluate the factors associated with 30-day mortality. The most common etiology for markedly elevated AST levels was skeletal muscle damage (54.2%), followed by cardiac muscle damage (39.1%) and hematologic disorder (6.7%). The 30-day mortality rates for the skeletal muscle damage, cardiac muscle damage, and hematologic disorder groups were 14.2%, 19.5%, and 65.5%, respectively. The magnitude of the peak AST level significantly correlated with 30-day mortality, with rates of 12.8%, 26.7%, and 50.0% for peak AST levels < 1000 U/L, <3000 U/L, and ≥3000 U/L, respectively. In the multivariate analysis, cardiac muscle damage (odds ratio [OR] = 2.76, 95% confidence interval [CI] = 1.31–5.80), hematologic disorder (OR = 9.47, 95% CI = 2.95–30.39), peak AST < 3000 U/L (OR = 2.94, 95% CI = 1.36–6.35), and peak AST ≥ 3000 U/L (OR = 9.61, 95% CI = 3.54–26.08) were associated with increased 30-day mortality. Our study revealed three etiologies of markedly elevated AST unrelated to liver disease and showed that etiology and peak AST level significantly affected the survival rate.

## 1. Introduction

Alanine aminotransferase (ALT) and aspartate aminotransferase (AST) are often referred to as liver function tests, but rather than being true measurements of hepatic function, the release of AST and ALT from hepatocytes into the bloodstream represents hepatocellular injury. ALT is more specific for hepatocellular injury because of its much higher concentration in the liver tissue than in other tissues. In contrast, AST is widely detected in various cells, such as hepatocytes, cardiac and skeletal myocytes, and erythrocytes. The relative activity of AST in various tissues and cells is as follows: 7800 in the heart, 7100 in the liver, 5000 in the skeletal muscle, 4500 in the kidney, 2500 in the brain, 1400 in the pancreas, 700 in the spleen, 500 in the lung, and 40 in erythrocytes [1]. Therefore, elevated serum AST levels can be observed in a wide spectrum of non-hepatic disorders, such as acute coronary syndrome, renal infarction, cerebral infarction, rhabdomyolysis, pulmonary embolism, and hemolysis [2,3].

Elevated aminotransferase levels due to non-hepatic causes are frequently encountered in clinical practice. Skeletal muscle damage (i.e., rhabdomyolysis) is one of the most common non-hepatic causes of elevated aminotransferase levels. The mortality rate of rhabdomyolysis was reported to be 3–9% in non-intensive care unit (ICU) patients and 7–46% in ICU patients [4,5,6,7,8,9,10]. Cardiac muscle damage, such as due to acute myocardial infarction and myocarditis, is also one of the common non-hepatic causes of elevated aminotransferases. In a previous Canadian study, AST elevation above the upper limit of normal (ULN) occurred in 85.6% of patients with ST-elevation myocardial infarction (STEMI), with markedly elevated AST (>10 times the ULN) occurring in 3.3% [11]. In other studies, markedly elevated AST levels occurred in 3–5% of patients with STEMI [12,13]. They also identified that elevated AST was associated with increased mortality in these patients. In patients who underwent coronary artery bypass graft surgery, an elevated postoperative AST level (>300 U/L) was an independent predictor of mortality [14]. Apart from muscle damage, elevated AST levels from hematologic disorders are also occasionally encountered in clinical practice. Several case series of AST elevations in malignant and non-malignant hematologic disorders have been reported [15,16,17,18].

It is well-known that marked elevation of aminotransferase levels is associated with higher mortality, as it usually occurs in the setting of severe hepatic injury, such as in ischemic hepatitis and septic shock in critically ill patients. However, there have been no reports on mortality in patients with non-hepatic causes of markedly elevated AST levels, such as skeletal muscle damage, cardiac muscle damage, or hematologic disorders. Therefore, our study aimed to determine the non-hepatic etiologies of markedly elevated AST levels and investigate the factors associated with mortality in these cases.

## 2. Materials and Methods

### 2.1. Study Population

**Figure 1:**
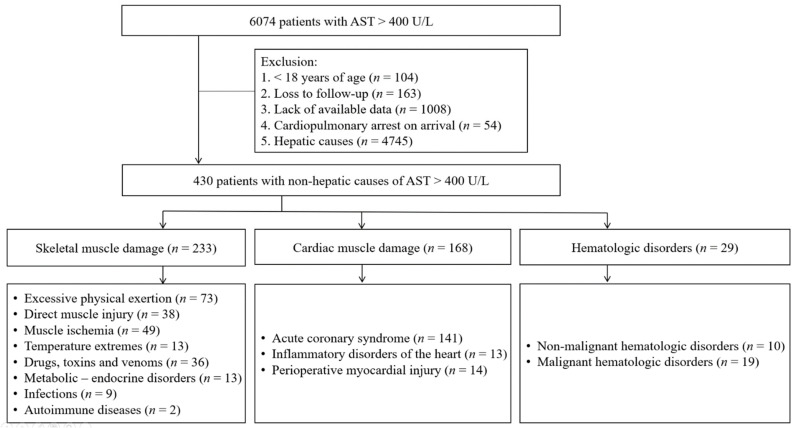
Flowchart of patients divided into three etiologic groups.

A total of 6074 consecutive patients with markedly elevated AST levels (defined as AST > 400 U/L) due to non-hepatic causes were identified from two centers between January 2010 and December 2021. The exclusion criteria were as follows: (1) age < 18 years (*n* = 104), (2) loss to follow-up (*n* = 163), (3) lack of available data (*n* = 1008), (4) cardiopulmonary arrest on arrival (*n* = 54), and hepatobiliary causes (*n* = 4745). The participants of this study included 430 patients with at least one instance of markedly elevated AST > 400 U/L and no evidence of liver disease (Figure 1). If multiple episodes of AST > 400 U/L occurred in one patient during the study period, we selected only the first episode for convenience of analysis.

### 2.2. Data Collection

Baseline demographics, clinical data, and laboratory data were retrieved from the patients’ medical records. Data on baseline demographics included age, sex, and underlying diseases, such as diabetes, liver cirrhosis, congestive heart failure, and end-stage renal disease. Clinical data included the presence of jaundice, infection, hypotension, need for renal replacement therapy, and hepatic decompensation, defined as the presence of jaundice (bilirubin > 12 mg/dL), hepatic encephalopathy, hepatorenal syndrome, ascites, or documented gastroesophageal variceal hemorrhage. Laboratory data included initial levels of AST, ALT, total bilirubin, direct bilirubin, albumin, alkaline phosphatase, creatine kinase (CK), lactate dehydrogenase, prothrombin time-international normalized ratio (PT-INR), and creatinine, as well as the peak levels of AST, ALT, and total bilirubin.

The index date for the analysis of the initial laboratory data was defined as the first date wherein an AST level > 400 U/L was reached. Serial AST, ALT, and bilirubin levels were measured at least every 1–2 days. The highest AST level within 30 days of the index date was defined as the peak AST level. Three grades for the severity of AST elevation were applied based on the peak AST level: (1) peak AST levels < 1000 U/L (as 400 U/L ≤ AST < 1000 U/L), (2) peak AST levels < 3000 U/L (as 1000 U/L ≤ AST < 3000 U/L), and (3) peak AST levels ≥ 3000 U/L.

### 2.3. Definitions and Classifications

Patients with markedly elevated AST levels due to hepatobiliary causes were excluded from the study. Hepatobiliary causes included the following: (1) pancreatobiliary diseases, including cholangitis, cholecystitis, and pancreatitis; (2) hepatocellular diseases, including drug-induced liver injury, viral hepatitis, autoimmune hepatitis, pregnancy-related liver disorder, alcoholic liver disease, liver involvement in systemic infection, and acute hepatitis of unknown origin; (3) liver surgery or liver trauma; (4) hepatobiliary malignancy; and (5) ischemic hepatitis. Patients with markedly elevated AST levels due to non-hepatobiliary causes were classified into three groups according to etiology: (1) skeletal muscle damage, (2) cardiac muscle damage, and (3) hematologic disorder (Figure 1).

#### 2.3.1. Skeletal Muscle Damage

Skeletal muscle damage (i.e., rhabdomyolysis) was defined as a serum CK level > 1000 U/L in the presence of skeletal muscle injury. We excluded patients with an insufficient CK profile, peak CK < 1000 U/L, or CK elevation due to myocardial muscle damage. We also excluded patients with elevated CK and AST levels due to concurrent ischemic hepatitis, as ischemic hepatitis resulting from hepatocellular injury is considered to have a greater effect on mortality than skeletal muscle damage. Skeletal muscle damage was stratified into eight causes: (1) excessive physical exertion, including exercise and seizures; (2) direct muscle injury, including crush injuries and burn; (3) muscle ischemia, including generalized ischemia, shock, emboli, thrombus, vascular occlusion, immobilization, and compartment syndrome; (4) temperature extremes, including heat stroke and hypothermia; (5) drugs, toxins, and venom, including alcohol use, benzodiazepine use, statin use, and snake bite; (6) metabolic and endocrine disorders, including hypothyroidism, hypokalemia, hyponatremia, hypernatremia, and diabetic ketoacidosis; (7) infections, including viral, bacterial, and parasitic etiologies; and (8) autoimmune diseases, including polymyositis and dermatomyositis.

#### 2.3.2. Cardiac Muscle Damage

Cardiac muscle damage was defined as a serum CK level > 500 U/L in the presence of cardiac muscle injury. We excluded patients with an insufficient CK profile, peak CK level < 500 U/L, CK elevation due to skeletal muscle damage, and concurrent ischemic hepatitis (defined as sharp increase in aminotransferase > 400 U/L exclusion of other causes of acute liver injury, and in an appropriate clinical setting of cardiac, respiratory, and circulatory failure, and septic shock). Through a retrospective review, patients with cardiac muscle damage and markedly elevated AST were classified into three categories: (1) acute coronary syndrome, including STEMI and non-STEMI, and unstable angina, (2) inflammatory disorders of the heart, including endocarditis, myocarditis, and pericarditis, and (3) perioperative myocardial injury (within 5 days postoperatively).

#### 2.3.3. Hematologic Disorder

Patients with an elevation of AST levels associated with disorders of the blood and blood-forming organs were classified under the hematologic disorder group. We excluded patients with elevated AST levels due to concurrent ischemic hepatitis but included patients with hepatic involvement in hematologic malignancy. Hematologic disorders were classified as (1) malignant, such as leukemia, lymphoma, and multiple myeloma; and (2) non-malignant, such as paroxysmal nocturnal hemoglobinuria, thrombotic thrombocytopenic purpura, idiopathic myelofibrosis, myelodysplastic syndrome, and hemophagocytic lymphohistiocytosis.

### 2.4. Statistical Analysis

Clinical and laboratory characteristics of patients are expressed as numbers (%) for categorical data and medians (interquartile range) for continuous data. For categorical variables, between-group differences were compared using the Chi-squared test or Fisher’s exact test. For continuous variables, the Mann–Whitney U test and Kruskal–Wallis test were used as appropriate. Survival curves for 30-day mortality were constructed using the Kaplan–Meier method with the log-rank test. Univariate and multivariate binary logistic regression analyses were performed to evaluate the odds ratios (ORs) with 95% confidence intervals (CIs) for 30-day mortality. The accuracy of the initial AST, initial ALT, peak AST, and peak ALT in correlating with 30-day mortality was assessed by the area under the receiver operating characteristic curve. A two-sided *p* value < 0.05 was considered statistically significant for all analyses. The PASW software (version 18; SPSS Inc., Chicago, IL, USA) and R software (version 3.4.1; R Foundation for Statistical Computing, Vienna, Austria) were used to process the data.

### 2.5. Ethics Statement

The present study protocol was reviewed and approved by the institutional review boards of Gyeongsang National University Changwon Hospital (IRB File No. 2021-06-032) and Gyeongsang National University Hospital (IRB File No. 2015-07-029). The requirement for informed consent was waived owing to the retrospective design of this study, as determined by the institutional review boards.

## 3. Results

### 3.1. Patient Characteristics

The baseline characteristics of 430 subjects are provided in Table 1, among whom 86 were non-survivors and 344 were survivors at day 30. The median age of the patients was 60.5 years and 309 patients were male (71.9%). At enrollment, 21 (4.9%) patients had liver cirrhosis, 58 (13.5%) had diabetes, 21 (4.9%) had congestive heart failure, and 5 (1.2%) had end-stage renal disease. The median initial AST and ALT levels were 657.0 U/L and 207.5 U/L, respectively. The median peak AST level was 782.0 U/L.

### 3.2. Clinical Outcomes

At enrollment, we identified 325 (75.6%) patients with initial AST levels < 1000 U/L, 94 (21.9%) with initial AST levels < 3000 U/L, and 11 (2.6%) with initial AST levels ≥ 3000 U/L. During the follow-up period, 274 (63.7%) patients had a peak AST level < 1000 U/L, 116 (27.0%) had a peak AST level < 3000 U/L, and 40 (9.3%) had a peak AST level ≥ 3000 U/L. A total of 81 (18.8%) patients with markedly elevated AST levels developed jaundice during the study period. Of the 430 patients, 112 (26.0%) were admitted to the ICU and 13 (3.0%) had hepatic decompensation at enrollment. In the entire study group, the 30-day mortality rate was 20%.

### 3.3. Non-Hepatic Etiologies of Elevated AST

**Figure 2:**
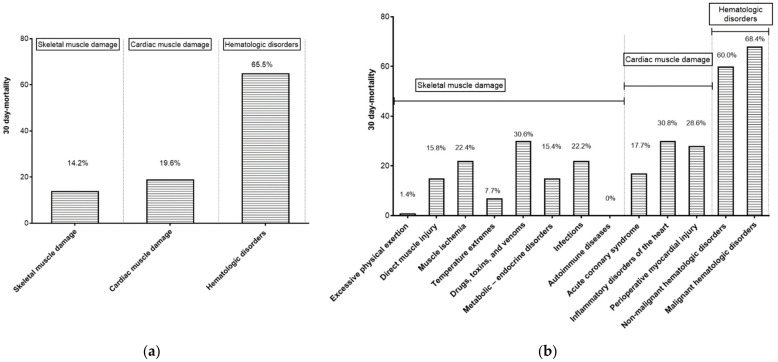
Thirty-day mortality rates of the 3 etiologic groups (**a**) and 13 subdivided categories (**b**).

The three groups of patients are shown in Table 2. The group with the most participants was the skeletal muscle damage group (*n* = 233, 54.2%), followed by the cardiac muscle damage group (*n* = 168, 39.1%) and the hematologic disorder group (*n* = 29, 6.7%). Patients in the skeletal muscle damage group had the highest initial and peak AST levels among the groups. The cardiac muscle damage group had the highest number of patients with diabetes (20.8%) and pre-existing congestive heart failure (8.9%), as well as patients admitted to the ICU (41.1%). The hematologic disorder group had the most patients with hepatic decompensation (13.8%), infection (48.3%), and hypotension (48.3%). The 30-day mortality rates were 14.2%, 19.6%, and 65.5% in the skeletal muscle damage, cardiac muscle damage, and hematologic disorder group, respectively (Figure 2a).

#### 3.3.1. Skeletal Muscle Damage Group

The most common cause of skeletal muscle damage in this group was excessive physical exertion (*n* = 73), followed by muscle ischemia (*n* = 49), direct muscle injury (*n* = 38), and drugs, toxins, and venom (*n* = 36) (Figure 1). Among these, drugs, toxins, and venom had the highest 30-day mortality rate (30.6%) (Figure 2b).

#### 3.3.2. Cardiac Muscle Damage Group

The most common cause of cardiac muscle damage was acute coronary syndrome (*n* = 141), followed by perioperative myocardial injury (*n* = 14) and inflammatory disorders of the heart (*n* = 13). Among these, inflammatory disorders of the heart had the highest 30-day mortality rate (30.8%).

#### 3.3.3. Hematologic Disorder Group

The hematologic disorder group was divided into malignant hematologic disorders (*n* = 19), including leukemia (*n* = 4), lymphoma (*n* = 8), and multiple myeloma (*n* = 7), and non-malignant hematologic disorders (*n* = 10), including paroxysmal nocturnal hemoglobinuria (*n* = 2), thrombotic thrombocytopenic purpura (*n* = 1), idiopathic myelofibrosis (*n* = 1), myelodysplastic syndrome (*n* = 2), and hemophagocytic lymphohistiocytosis (*n* = 4). Malignant and non-malignant hematologic disorders had 30-day mortality rates of 68.4% and 60%, respectively.

### 3.4. Risk Factors Associated with 30-Day Mortality

**Figure 3:**
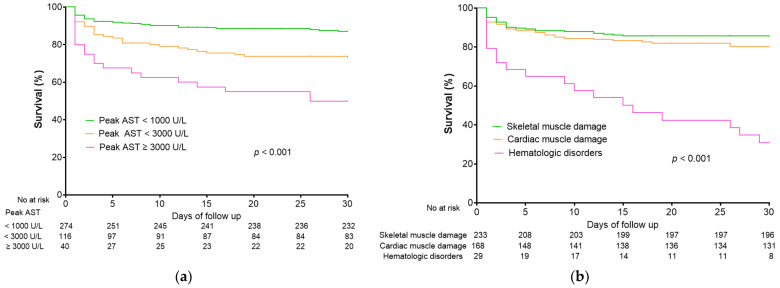
Kaplan–Meier curve for survival according to (**a**) peak AST level and (**b**) etiologic group.

The area under the receiver operating characteristic curve of the initial AST, initial ALT, peak AST, and peak ALT were 0.558 (95% CI, 0.490–0.626), 0.580 (95% CI, 0.510–0.650), 0.686 (95% CI, 0.621–0.751), and 0.674 (95% CI, 0.609–0.738), respectively (Supplementary Figure S1). Peak AST levels were strongly correlated with 30-day mortality, with mortality rates of 12.8%, 26.7%, and 50.0% for patients with peak AST levels < 1000 U/L, <3000 U/L, and ≥3000 U/L, respectively (Figure 3a). Patients with hematologic disorders had a higher 30-day mortality rate (65.5%) than those with cardiac muscle damage (19.6%, *p* < 0.001) and skeletal muscle damage (14.2%, *p* < 0.001) (Figure 3b).

The skeletal muscle group was used as a reference for the etiologic groups while peak AST levels < 1000 IU/L was used as a reference for peak AST levels in the logistic regression analyses. In the univariate analysis, age (OR = 1.04 per year, 95% CI = 1.03–1.06), hepatic decompensation (OR = 6.95, 95% CI = 2.22–21.83), albumin level (OR = 0.22, 95% CI = 0.15–0.33), bilirubin level (OR = 1.22, 95% CI = 1.10–1.36), creatinine level (OR = 1.87, 95% CI = 1.52–2.30), PT-INR (OR = 3.35, 95% CI = 2.15–5.23), hematologic disorder (OR = 11.12, 95% CI = 4.76–25.96), peak AST < 3000 U/L (OR = 2.49, 95% CI = 1.45–4.29), and peak AST ≥ 3000 U/L (OR = 6.83, 95% CI = 3.34–13.95) were associated with 30-day mortality. In the multivariate analysis, age (adjusted OR = 1.04 per year, 95% CI = 1.02–1.06), albumin level (adjusted OR = 0.28, 95% CI = 0.16–0.50), creatinine level (adjusted OR = 1.81, 95% CI = 1.42–2.31), cardiac muscle damage (adjusted OR = 2.76, 95% CI = 1.31–5.80), hematologic disorder (adjusted OR = 9.47, 95% CI = 2.95–30.39), peak AST < 3000 U/L (adjusted OR = 2.94, 95% CI = 1.36–6.35), and peak AST ≥ 3000 U/L (adjusted OR = 9.61, 95% CI = 3.54–26.08) were independently associated with 30-day mortality (Table 3).

### 3.5. Impact of Etiology and Peak AST Levels on Mortality

**Figure 4:**
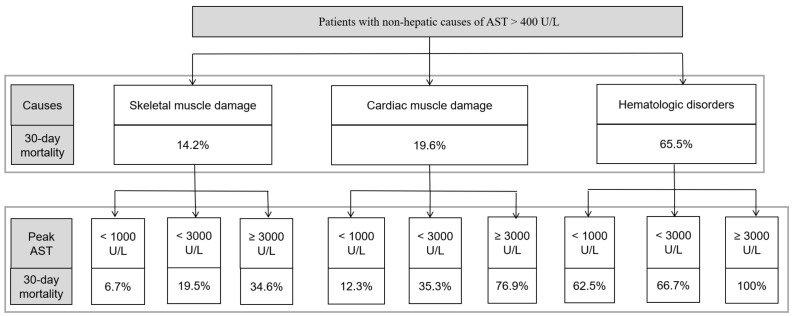
Simplified algorithm based on etiologies and peak AST.

In patients with markedly elevated AST levels unrelated to liver disease, we developed a simplified algorithm that incorporates etiology and peak AST levels (Figure 4). First, a non-hepatic cause should be established in all patients with AST levels > 400 U/L. With this, the estimated 30-day mortality rates are 14.2%, 19.6%, and 65.5% in the skeletal muscle damage, cardiac muscle damage, and hematologic disorder group, respectively. Second, each etiological group was stratified according to peak AST levels during follow-up testing. For example, in the skeletal muscle damage group, the 30-day mortality rate in patients with peak AST levels < 1000 U/L was 6.7%, while the 30-day mortality rate of those with peak AST levels ≥ 3000 U/L was 34.6%.

## 4. Discussion

In this study, we found that the most common non-hepatic cause of markedly elevated AST levels was skeletal muscle damage (54.2%), followed by cardiac muscle damage (39.1%) and hematologic disorders (6.7%). Patients with markedly elevated AST levels unrelated to liver disease had a 30-day mortality rate of 20%. The magnitude of the peak AST levels and etiology of elevated AST levels were independent risk factors of 30-day mortality.

AST activity has been widely detected in various human tissues. It is generally thought that increased levels of circulating AST may reflect direct tissue damage and apoptosis related to liver inflammation, septic shock, acute myocardial infarction, and skeletal muscle injury [19]. In fact, plasma membrane blebs containing cytosolic components have been demonstrated during ischemia-reperfusion injuries in the myocardium and liver [20]. Skeletal muscle is known to contain intracellular proteins such as myoglobin and isozymes such as CK, lactate dehydrogenase, AST, and ALT which may be released into the circulation following muscle injury [21]. Elevated AST levels due to cardiac muscle damage may thus be explained by the release of AST from the myocardium due to increased stress, acute ischemia and necrosis in acute coronary syndrome and reperfusion injury [22]. Lastly, hepatic infiltration by malignant hematologic disorders, such as leukemia, lymphoma, and multiple myeloma, and non-malignant hematologic disorders, such as idiopathic myelofibrosis, hemolytic anemia, polycythemia vera, myelodysplastic syndrome, and hemophagocytic lymphohistiocytosis, may also cause AST elevation [17,18,23]. Additionally, hemolysis, iron deposition in the liver, repeated transfusions, concomitant infection, and disseminated intravascular coagulation may contribute to elevated AST in patients with this group [15,24].

We identified the three main non-hepatic etiologies of markedly elevated AST levels and demonstrated that etiology affects patient survival. To the best of our knowledge, there have been no studies on the prognosis of patients with markedly elevated AST levels unrelated to liver disease. In previous studies, mortality rates in patients with rhabdomyolysis ranged from 3% to 46% depending on the study population, frequency of acute kidney injury, comorbidities, and cause of rhabdomyolysis [4,5,6,7,8,9,10]. A previous study by Weibrecht et al. reported that elevated AST (>40 U/L) occurred in 93% of patients with rhabdomyolysis [7]. Raurich et al. reported that the incidence of elevated aminotransferases > 1000 U/L in ICU patients with rhabdomyolysis was 24.3%. Moreover, they found that patients with rhabdomyolysis-related elevated aminotransferase levels (>1000 U/L) had higher mortality than patients with low aminotransferase levels (69.4% vs. 14.7%) [5]. A previous study by Lofthus et al. reported that elevated AST levels above the ULN were common (occurring in 85%) in the setting of STEMI. Other studies have reported that the incidence of markedly elevated AST (>400 U/L) in patients with STEMI was 3–5%. They also found that patients with markedly elevated AST had a higher mortality rate than those without markedly elevated AST [11,12,13]. In this study, we found that the 30-day mortality rate in patients with markedly elevated AST not due to liver disease was 14.2%, 19.6%, and 65.5% for skeletal muscle damage, cardiac muscle damage, and hematologic disorders, respectively.

In the present study, we arbitrarily used a cutoff level of 400 U/L for markedly elevated AST levels. Previous studies on patients with markedly elevated aminotransferase levels due to liver disease showed that all-cause mortality rate correlated with increasing aminotransferase levels. The reported all-cause mortality rates were 16–22%, 30–45%, and 31–55% in patients with aminotransferase levels > 400 U/L [25,26,27], >1000 U/L [27,28,29], and >3000 U/L, respectively [30,31]. Our study showed that the magnitude of peak AST levels during the course of extrahepatic injury significantly correlated with 30-day mortality, with rates of 12.8%, 26.7%, and 50.0% in patients with peak AST levels < 1000 U/L, <3000 U/L, and ≥3000 U/L, respectively. In the multivariate analysis, etiology, peak AST levels, age, albumin level, and creatinine level were independent factors associated with mortality. In contrast, factors reflecting liver function, including hepatic decompensation, bilirubin level, ALT level, and PT-INR, were not found to be associated with mortality.

In clinical practice, patients with markedly elevated aminotransferase levels with no evidence of liver disease present a significant challenge to physicians due to their high mortality. In this study, among 5644 patients with AST > 400 U/L, only 7.6% were found to be associated with non-hepatic causes. This suggests that markedly elevated AST levels unrelated to liver disease may not be encountered frequently in clinical practice. Therefore, we present a simplified schematic approach for patients with markedly elevated AST levels unrelated to liver disease, involving (1) determination of the etiology (skeletal muscle damage, cardiac muscle damage, or hematologic disorder) and (2) measurement of the peak AST level during follow-up testing (<1000 U/L, <3000 U/L, or ≥3000 U/L) (Figure 4).

One limitation of this study is that the etiology of markedly elevated AST levels could not be determined prospectively. Another limitation is that additional liver damage, including ischemic injury, multiple organ failure, and drug-induced liver injury, may have contributed to the magnitude of AST levels during follow-up testing, although there was no evidence of liver disease at enrollment in all patients. In addition, we only focused on 30-day mortality in patients with markedly elevated AST. Therefore, further prospective study is needed to elucidate the factors that affect the long-term prognosis of patients who survived more than 30 days. Nevertheless, the strength of this study is that it is the first to examine the etiologies and prognoses of markedly elevated AST levels in patients without liver disease.

## 5. Conclusions

Various etiologies affect the survival rate of patients with markedly elevated AST levels (>400 U/L) unrelated to liver disease. Etiology and peak AST levels, but not factors reflecting liver function (bilirubin, ALT, and PT-INR), were independent factors associated with mortality in these patients. Large prospective studies are required to further elucidate the etiologies and prognoses of markedly elevated AST levels in this patient group.

## Figures and Tables

**Table 1:** Clinical and laboratory characteristics of patients (*n* = 430).

Characteristics	Initial AST < 657 U/L	Initial AST ≥ 657 U/L	Total
No.	215	215	430
Age, year	61.0 (41.0–72.0)	61.0 (46.0–74.0)	60.5 (42.8–72.3)
Male gender	154 (71.6%)	155 (72.1%)	309 (71.9%)
Causes			
Skeletal muscle damage	84 (36.1%)	149 (63.9%)	233 (54.2%)
Cardiac muscle damage	117 (69.6%)	51 (30.4%)	168 (39.1%)
Hematologic disorders	14 (48.3%)	15 (51.7%)	29 (6.7%)
Liver cirrhosis	5 (2.3%)	16 (7.4%)	21 (4.9%)
Diabetes	33 (15.3%)	25 (11.6%)	58 (13.5%)
CHF	11 (5.1%)	10 (4.7%)	21 (4.9%)
ESRD	2 (0.9%)	3 (1.4%)	5 (1.2%)
Hepatic decompensation	6 (2.8%)	7 (3.3%)	13 (3.0%)
Infection	23 (10.7%)	42 (19.5%)	65 (15.1%)
Hypotension	53 (24.7%)	61 (28.4%)	114 (26.5%)
Admission at ICU	54 (25.1%)	58 (27.0%)	112 (26.0%)
Initial values			
AST, U/L	527.0 (477.0–579.0)	983.0 (775.0–1473.0)	657.0 (527.0–984.3)
ALT, U/L	141.0 (101.0–219.0)	340.0 (189.0–599.0)	207.5 (126.8–401.8)
Albumin, g/dL	3.5 (2.9–4.0)	3.6 (3.0–4.1)	3.5 (3.0–4.0)
Bilirubin, mg/dL	1.06 (0.72–1.62)	1.02 (0.73–1.62)	1.03 (0.73–1.62)
ALP, U/L	73.0 (58.0–97.0)	74.0 (57.0–103.0)	73.0 (58.0–100.0)
LDH, U/L	910.0 (495.8–1197.0)	1676.0 (973.0–2700.0)	1148.0 (716.5–1951.0)
CK, U/L	4294.0 (3276.0–14,503.0)	9250.0 (3301.5–45,757.0)	5646.0 (3293.8–25,000.0)
Creatinine, mg/dL	0.94 (0.70–1.47)	1.20 (0.85–1.91)	1.05 (0.76–1.63)
PT-INR	1.05 (0.99–1.28)	1.17 (1.02–1.64)	1.11 (1.00–1.38)
Peak values	168.0 (111.0–310.0)	462.0 (250.0–927.0)	264.0 (147.8–572.0)
AST, U/L	559.0 (496.0–639.0)	846.0 (1198.0–2253.0)	782.0 (559.0–1400.3)
ALT, U/L			
Bilirubin, mg/dL	1.30 (0.88–2.50)	1.30 (0.85–2.25)	1.30 (0.86–2.39)

Abbreviation: CHF, congestive heart failure; ESRD, end-stage renal disease; ICU, intensive care unit; AST, aspartate aminotransferase; ALT, alanine aminotransferase; ALP, Alkaline Phosphatase; LDH, lactate dehydrogenase; CK, creatine kinase; PT-INR, prothrombin time-international normalized ratio. P: The Mann–Whitney U test and Chi-squared test. Data are presented as medians (interquartile range) for continuous data and as percentages for categorical data.

**Table 2:** Clinical and laboratory characteristics of patients according to etiology (*n* = 430).

Characteristics	Skeletal Muscle Damage	Cardiac Muscle Damage	Hematologic Disorders	*p*
No.	233 (54.2%)	168 (39.1%)	29 (6.7%)	
Age, year	49.0 (34.0–65.5)	66.0 (56.0–76.0)	70.0 (58.0–79.5)	<0.001
Male gender	168 (72.1%)	125 (74.4%)	16 (55.2%)	0.103
Liver cirrhosis	18 (7.7%)	3 (1.8%)	0 (0%)	0.011
Diabetes	21 (9.0%)	35 (20.8%)	2 (6.9%)	0.002
CHF	4 (1.7%)	15 (8.9%)	2 (6.9%)	0.004
ESRD	3 (1.3%)	2 (1.2%)	0 (0%)	0.830
Hepatic decompensation	7 (3.0%)	2 (1.2%)	4 (13.8%)	0.001
Infection	32 (13.7%)	19 (11.3%)	14 (48.3%)	<0.001
Hypotension	46 (19.7%)	54 (32.1%)	14 (48.3%)	<0.001
Admission at ICU	37 (15.9%)	69 (41.1%)	6 (20.7%)	<0.001
Initial values				
AST, U/L	765.0 (562.0–1311.0)	568.0 (502.0–711.5)	663.0 (504.0–928.5)	<0.001
ALT, U/L	252.0 (168.0–477.0)	139.0 (100.3–251.5)	266.0 (146.0–501.5)	<0.001
Albumin, g/dL	3.7 (3.0–4.3)	3.5 (3.2–3.8)	2.9 (2.3–3.5)	<0.001
Bilirubin, mg/dL	1.00 (0.71–1.58)	0.99 (0.73–1.42)	2.62 (1.17–8.25)	<0.001
ALP, U/L	72.0 (57.0–97.5)	71.0 (58.0–93.0)	113.0 (76.5–238.5)	<0.001
LDH, U/L	1383.0 (866.5–2242.3)	854.5 (285.3–1343.3)	2318.0 (1143.5–2853.0)	<0.001
CK, U/L	24,000.0 (5922.5–27,750.0)	4100.0 (2321.3–5220.3)	71.0 (34.0–459.0)	<0.001
Creatinine, mg/dL	1.03 (0.74–1.89)	1.05 (0.76–1.50)	1.44 (0.84–2.15)	0.229
PT-INR	1.15 (1.00–1.39)	1.04 (0.99–1.24)	1.42 (1.19–1.86)	<0.001
Peak values				
AST, U/L	960.0 (619.5–1757.0)	624.5 (518.0–821.8)	951.0 (679.0–1300.5)	<0.001
ALT, U/L	361.0 (206.5–658.5)	155.0 (105.5–339.5)	345.0 (166.5–595.0)	<0.001
Bilirubin, mg/dL	1.27 (0.83–2.42)	1.24 (0.87–1.99)	4.28 (2.27–20.60)	<0.001

Abbreviation: CHF, congestive heart failure; ESRD, end-stage renal disease; ICU, intensive care unit; AST, aspartate aminotransferase; ALT, alanine aminotransferase; ALP, Alkaline Phosphatase; LDH, lactate dehydrogenase; CK, creatine kinase; PT-INR, prothrombin time- international normalized ratio. P: The Kruskal–Wallis test and Chi-squared test. Data are presented as medians (interquartile ranges) for continuous data and as percentages for categorical data.

**Table 3:** Risk factors of 30-day mortality in univariate and multivariate logistic regression analyses (*n* = 430).

Variable	Univariate Analysis		Multivariate Analysis	
	*p*	OR (95% CI)	*p*	OR (95% CI)
Male	0.07	0.63 (0.38–1.04)		
Age, per year	<0.001	1.04 (1.03–1.06)	0.001	1.04 (1.02–1.06)
Etiology				
Skeletal muscle damage		Reference		Reference
Cardiac muscle damage	0.182	1.43 (0.85–2.42)	0.008	2.76 (1.31–5.80)
Hematologic disorder	<0.001	11.12 (4.76–25.96)	<0.001	9.47 (2.95–30.39)
Hepatic decompensation	0.001	6.95 (2.22–21.83)	0.462	1.80 (0.38–8.56)
Albumin, g/dL	<0.001	0.22 (0.15–0.33)	<0.001	0.28 (0.16–0.50)
Bilirubin, mg/dL	<0.001	1.22 (1.10–1.36)	0.190	1.09 (0.96–1.23)
Creatinine, mg/dL	<0.001	1.87 (1.52–2.30)	<0.001	1.81 (1.42–2.31)
PT-INR	<0.001	3.35 (2.15–5.23)	0.591	1.17 (0.67–2.03)
Peak AST				
<1000 U/L		Reference		Reference
<3000 U/L	0.001	2.49 (1.45–4.29)	0.006	2.94 (1.36–6.35)
≥3000 U/L	<0.001	6.83 (3.34–13.95)	<0.001	9.61 (3.54–26.08)

Abbreviation: OR, odds ratio; CI, confidence interval; PT-INR, prothrombin time- international normalized ratio; AST, aspartate aminotransferase.

## Data Availability

The datasets generated and/or analyzed during the current study are not publicly available due to ethical and confidentiality reasons but are available from the corresponding author on reasonable request under the Gyeongsang National University Changwon Hospital and Gyeongsang National University Hospital Ethics Committee’s approval. The data that support the findings of this study are available on request to the correspondence author.

## Supplementary Materials

The following supporting information can be downloaded at: https://www.mdpi.com/article/10.3390/jcm12010310/s1, Figure S1: The area under the receiver operating characteristic curve of the initial AST, initial ALT, peak AST, and peak ALT in correlating with 30-day mortality. *p* = 0.469 for initial AST vs initial ALT, *p* = 0.632 for peak AST vs peak ALT, *p* < 0.001 for peak AST vs initial AST, and *p* = 0.004 for peak AST vs initial ALT.

## References

[1] Botros M., Sikaris K.A. (2013). The de ritis ratio: The test of time. Clin. Biochem. Rev..

[2] Kwo P.Y., Cohen S.M., Lim J.K. (2017). ACG Clinical Guideline: Evaluation of Abnormal Liver Chemistries. Am. J. Gastroenterol..

[3] Giannini E.G., Testa R., Savarino V. (2005). Liver enzyme alteration: A guide for clinicians. CMAJ.

[4] Lim A.K.H., Arumugananthan C., Lau Hing Yim C., Jellie L.J., Wong E.W.W., Junckerstorff R.K. (2019). A Cross-Sectional Study of the Relationship between Serum Creatine Kinase and Liver Biochemistry in Patients with Rhabdomyolysis. J. Clin. Med..

[5] Raurich J.M., Llompart-Pou J.A., Rodriguez-Yago M., Ferreruela M., Royo C., Ayestaran I. (2015). Role of Elevated Aminotransferases in ICU Patients with Rhabdomyolysis. Am. Surg..

[6] El-Abdellati E., Eyselbergs M., Sirimsi H., Hoof V.V., Wouters K., Verbrugghe W., Jorens P.G. (2013). An observational study on rhabdomyolysis in the intensive care unit. Exploring its risk factors and main complication: Acute kidney injury. Ann. Intensive Care.

[7] Weibrecht K., Dayno M., Darling C., Bird S.B. (2010). Liver aminotransferases are elevated with rhabdomyolysis in the absence of significant liver injury. J. Med. Toxicol..

[8] Melli G., Chaudhry V., Cornblath D.R. (2005). Rhabdomyolysis: An evaluation of 475 hospitalized patients. Medicine.

[9] de Meijer A.R., Fikkers B.G., de Keijzer M.H., van Engelen B.G., Drenth J.P. (2003). Serum creatine kinase as predictor of clinical course in rhabdomyolysis: A 5-year intensive care survey. Intensive Care Med..

[10] Vangstad M., Bjornaas M.A., Jacobsen D. (2019). Rhabdomyolysis: A 10-year retrospective study of patients treated in a medical department. Eur. J. Emerg. Med..

[11] Lofthus D.M., Stevens S.R., Armstrong P.W., Granger C.B., Mahaffey K.W. (2012). Pattern of liver enzyme elevations in acute ST-elevation myocardial infarction. Coron. Artery Dis..

[12] Moon J., Kang W., Oh P.C., Seo S.Y., Lee K., Han S.H., Ahn T., Shin E. (2014). Serum transaminase determined in the emergency room predicts outcomes in patients with acute ST-segment elevation myocardial infarction who undergo primary percutaneous coronary intervention. Int. J. Cardiol..

[13] Gao M., Cheng Y., Zheng Y., Zhang W., Wang L., Qin L. (2017). Association of serum transaminases with short- and long-term outcomes in patients with ST-elevation myocardial infarction undergoing primary percutaneous coronary intervention. BMC Cardiovasc. Disord..

[14] van Boxtel A.G., Bramer S., Soliman Hamad M.A., van Straten A.H. (2012). Perioperative serum aspartate aminotransferase level as a predictor of survival after coronary artery bypass grafting. Ann. Thorac. Surg..

[15] Singh M.M., Pockros P.J. (2011). Hematologic and oncologic diseases and the liver. Clin. Liver. Dis..

[16] Murakami J., Shimizu Y. (2013). Hepatic manifestations in hematological disorders. Int. J. Hepatol..

[17] Baumhoer D., Tzankov A., Dirnhofer S., Tornillo L., Terracciano L.M. (2008). Patterns of liver infiltration in lymphoproliferative disease. Histopathology.

[18] Walz-Mattmuller R., Horny H.P., Ruck P., Kaiserling E. (1998). Incidence and pattern of liver involvement in haematological malignancies. Pathol. Res. Pract..

[19] McGill M.R. (2016). The past and present of serum aminotransferases and the future of liver injury biomarkers. EXCLI J..

[20] Gores G.J., Herman B., Lemasters J.J. (1990). Plasma membrane bleb formation and rupture: A common feature of hepatocellular injury. Hepatology.

[21] Lim A.K. (2020). Abnormal liver function tests associated with severe rhabdomyolysis. World J. Gastroenterol..

[22] Schwartz P., Piper H.M., Spahr R., Spieckermann P.G. (1984). Ultrastructure of cultured adult myocardial cells during anoxia and reoxygenation. Am. J. Pathol..

[23] Scheimberg I.B., Pollock D.J., Collins P.W., Doran H.M., Newland A.C., van der Walt J.D. (1995). Pathology of the liver in leukaemia and lymphoma. A study of 110 autopsies. Histopathology.

[24] Shimizu Y. (2008). Liver in systemic disease. World J. Gastroenterol..

[25] Whitehead M.W., Hawkes N.D., Hainsworth I., Kingham J.G. (1999). A prospective study of the causes of notably raised aspartate aminotransferase of liver origin. Gut.

[26] Bjornsson H.K., Olafsson S., Bergmann O.M., Bjornsson E.S. (2016). A prospective study on the causes of notably raised alanine aminotransferase (ALT). Scand. J. Gastroenterol..

[27] Van den Broecke A., Van Coile L., Decruyenaere A., Colpaert K., Benoit D., Van Vlierberghe H., Decruyenaere J. (2018). Epidemiology, causes, evolution and outcome in a single-center cohort of 1116 critically ill patients with hypoxic hepatitis. Ann. Intensive Care.

[28] Galvin Z., McDonough A., Ryan J., Stewart S. (2015). Blood alanine aminotransferase levels > 1000 IU/l-causes and outcomes. Clin. Med..

[29] Con D., Buckle A., Nicoll A.J., Lubel J.S. (2020). Epidemiology and outcomes of marked elevations of alanine aminotransferase >1000 IU/L in an Australian cohort. JGH Open.

[30] Johnson R.D., O’Connor M.L., Kerr R.M. (1995). Extreme serum elevations of aspartate aminotransferase. Am. J. Gastroenterol..

[31] Saito K., Sugawara H., Watanabe T., Ishii A., Fukuchi T. (2021). A retrospective cross-sectional study for predicting 72-h mortality in patients with serum aspartate aminotransferase levels >/= 3000 U/L. Sci. Rep..

