# Supplementary material for: Markedly Elevated Aspartate Aminotransferase from Non-Hepatic Causes
Source: J Clin Med. 2022 Dec 30;12(1):310. doi: 10.3390/jcm12010310 (PMC9821092; doi:10.3390/jcm12010310)
Supplement: Supplementary file 1 [file jcm-12-00310-s001.zip › jcm-2110660-supplementary.pdf]

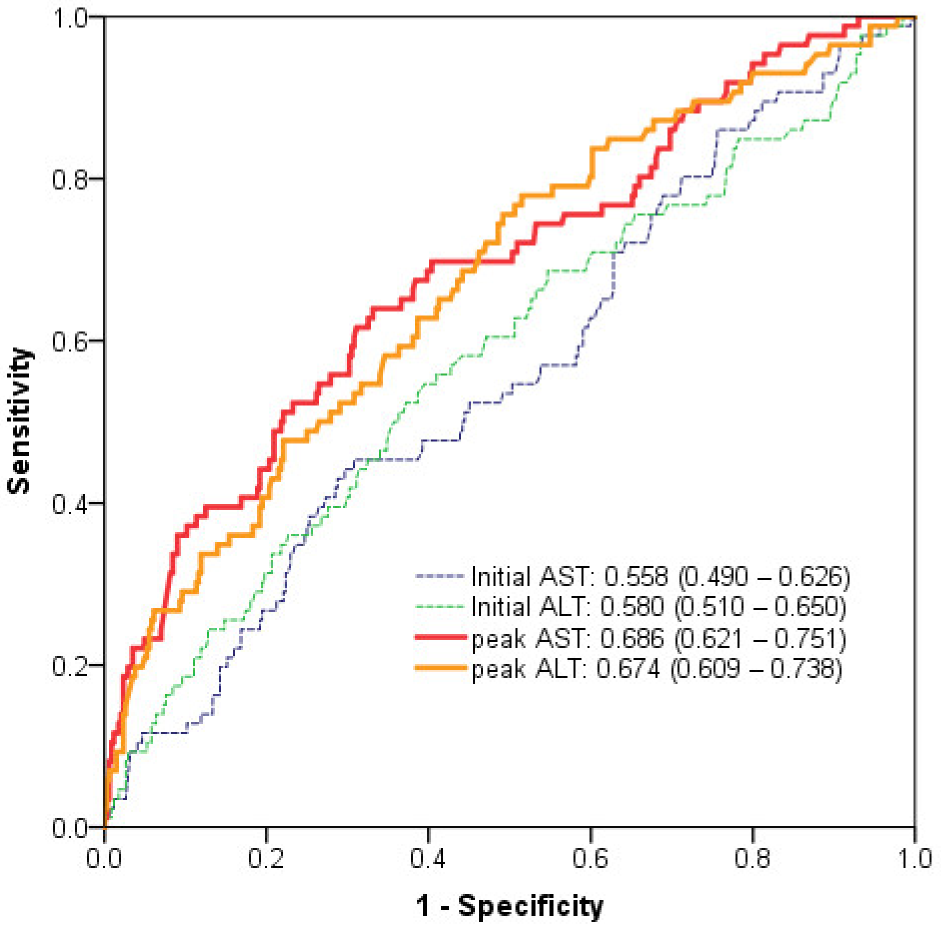

Figure S1. The area under the receiver operating characteristic curve of the initial AST, initial ALT, peak AST, and peak ALT in correlating with 30-day mortality. $p = 0.469$ for initial AST vs initial ALT, $p = 0.632$ for peak AST vs peak ALT, $p < 0.001$ for peak AST vs initial AST, and $p = 0.004$ for peak AST vs initial ALT.
